# Multi-Walled Carbon Nanotubes Can Promote *Brassica napus* L. and *Arabidopsis thaliana* L. Root Hair Development through Nitric Oxide and Ethylene Pathways

**DOI:** 10.3390/ijms21239109

**Published:** 2020-11-30

**Authors:** Gan Zhao, Yingying Zhao, Wang Lou, Dyaaaldin Abdalmegeed, Rongzhan Guan, Wenbiao Shen

**Affiliations:** 1 Laboratory Center of Life Sciences, College of Life Sciences, Nanjing Agricultural University, Nanjing 210095, China; 2018216033@njau.edu.cn (G.Z.); 2017116114@njau.edu.cn (Y.Z.); 2018116099@njau.edu.cn (W.L.); 2018116158@njau.edu.cn (D.A.); 2 State Key Laboratory of Crop Genetics and Germplasm Enhancement, Nanjing Agricultural University, Nanjing 210095, China; guanrzh@njau.edu.cn

**Keywords:** multi-walled carbon nanotubes, root hair, nitric oxide, ethylene

## Abstract

Here, we report that multi-walled carbon nanotubes (MWCNTs) can promote plant root hair growth in the species analyzed in this study; however, low and excessive concentrations of MWCNTs had no significant effect or even an inhibiting influence. Further results show that MWCNTs can enter rapeseed root cells. Meanwhile, nitrate reductase (NR)-dependent nitric oxide (NO) and ethylene syntheses, as well as root hair formation, were significantly stimulated by MWCNTs. Transcription of root hair growth-related genes were also modulated. The above responses were sensitive to the removal of endogenous NO or ethylene with a scavenger of NO or NO/ethylene synthesis inhibitors. Pharmacological and molecular evidence suggested that ethylene might act downstream of NR-dependent NO in MWCNTs-induced root hair morphogenesis. Genetic evidence in *Arabidopsis* further revealed that MWCNTs-triggered root hair growth was abolished in ethylene-insensitive mutants *ein2-5* and *ein3-1*, and NR mutant *nia1/2*, but not in *noa1* mutant. Further data placed NO synthesis linearly before ethylene production in root hair development triggered by MWCNTs. The above findings thus provide some insights into the molecular mechanism underlying MWCNTs control of root hair morphogenesis.

## 1. Introduction

In 1991, Iijima first produced carbon nanotubes (CNTs), which were confirmed with high-resolution transmission electron microscopy [1]. Subsequent work produced abundant single-shell tubes with diameters of about one nanometer [2]. Their radial and axial dimensions are both on the order of micrometers, and both ends of the tubes are open. Due to their special structures and unique physical, chemical, biological properties, and huge application potential, they have received considerable attention [3,4,5]. Because of their excellent transmembrane and adsorption capacity [6], the research on CNTs’ impact on agriculture is also emerging. Plants play vital roles in the ecological system; the edible parts of crops are possible routes for the uptake, translocation, and accumulation of CNTs, if they are used in agriculture [6,7]. In addition, CNTs can be toxic to animals and humans, mainly by inducing oxidative stress, mechanical damage, and affecting the activities of biological enzymes [8]. Therefore, to promote the application of CNTs in agriculture, the interactions between CNTs and plants need to be carefully elucidated.

Previous research on the relationship between CNTs and plants mainly focused on seed germination [9,10], primary root growth [11,12,13], lateral root development [14,15,16], flowering [17,18], and stress tolerance [19,20]. However, except for the studies of Joshi et al. [21] and García-Sánchez et al. [22], few investigations have been completed on the functions of multi-walled carbon nanotube (MWCNTs; one kind of CNTs) on root hairs and their corresponding mechanisms, and their phenotypes were controversial. Thus, the effects of CNTs on root hairs need to be further studied.

Root hair is one of the important components of root organogenesis. It could help the root system tightly integrating into the soil and increase the surface of the root system, thus helping plants successfully absorbing water and nutrients from the environment [23]. Also, root hair development can help plants grow out of trouble in a harmful environment [24].

During root hair development, ethylene is an important and essential signaling molecule [25,26,27,28]. ACC synthase and ACC oxidase, two critical enzymes responsible for ethylene biosynthesis, ubiquitously exist in plants [25,26,27]. Ethylene not only promotes the outgrowth of the root hair, but also induces the formation of root hairs on hairless cells [25,26]. These conclusions were based on the enhancement of root hair development observed in the presence of ethylene [26], 1-aminocyclopropane-1-carboxylic acid (ACC; an ethylene synthesis precursor), [27,28], and constitutive ethylene-responsive *ctr1* mutant [25]. Meanwhile, contrasting results were also confirmed in *ein2* [26,27], *ein3* [28], and *etr* [29], several ethylene-insensitive mutants, and in the wild type when exogenously applied with ethylene synthesis inhibitor cobalt chloride (CoCl_2_) [29,30] and aminoethoxyvinylglycine (AVG) [25,29].

It is well documented that nitric oxide (NO) in plants can be produced through two routes, enzymatic or nonenzymatic ones. Between these, nitrate reductase (NR) and nitric oxide synthase (NOS)-like protein are two important enzymes responsible for synthesizing NO [27,31]. Like ethylene, NO was also confirmed to be an important signal molecule in plant root hair development [31,32,33,34]. This conclusion was based on the pharmacological and genetic evidence, showing that exogenously applied with a NO-releasing compound sodium nitroprusside (SNP) favors *Arabidopsis* root hair growth [31,34]. Whereas the defective phenomenon was observed in *nia1/2* (impaired in nitrate reductase activity) [27,34] and *noa1* (encoding NO-associated protein 1; exhibiting a reduced endogenous NO level indirectly), two NO-related mutants [31,34], and in the wild type when supplemented with the NO scavenger 2-(4-carboxyphenyl)-4,4,5,5 -tetramethylimidazoline-1-oxyl-3-oxide (c-PTIO) [31,32], NR inhibitor tungstate, and NOS inhibitor *N*^G^-nitro-_L_-arginine methyl ester hydrochloride (_L_-NAME) [27].

During plant development, it has been shown that both ethylene and NO can act both synergistically (in particular synergistically) and independently [34,35,36]. For example, several data place ethylene synthesis linearly after NO signaling in cell wall phosphorus reutilization in P-deficient rice [35], and ethylene and NO work together to induce root hair development in *Arabidopsis* upon the magnesium deficiency conditions [27]. By contrast, NO is also required for cucumber adventitious root development elicited by ethylene [36]. However, no detailed evaluation has yet been carried out to assess the contribution of ethylene and NO in MWCNTs-triggered other plant responses.

Here, by using a pharmacological approach, the effects of various concentrations of MWCNTs on plant root hair growth were evaluated in rapeseed (*Brassica napus* L.), the third most important source of vegetable oil worldwide [37]. Our findings showed that MWCNTs could dose-dependently affect root hair development in terms of changes in root hair density and length. The distribution of MWCNTs was also evaluated. Because both rapeseed and *Arabidopsis* are classified as cruciferous plants and a high homolog exists in their genomes [38], *Arabidopsis ein2-5* and *ein3-1*, two ethylene-insensitive mutants, and *nia1/2* and *noa1*, two NO mutants, were utilized to investigate the relationship between NO and ethylene. The evidence further revealed the important key roles of both ethylene and NO in MWCNTs-triggered root hair development, and ethylene may act downstream of NR-dependent NO signaling. The above results provided a unique mechanism for NO-ethylene interaction during root hair development triggered by MWCNTs, and presented a theoretical basis for the possible application of CNTs in agriculture.

## 2. Results

### 2.1. MWCNTs-Stimulated Root Hair Growth and the Distribution of MWCNTs

**Figure 1:**
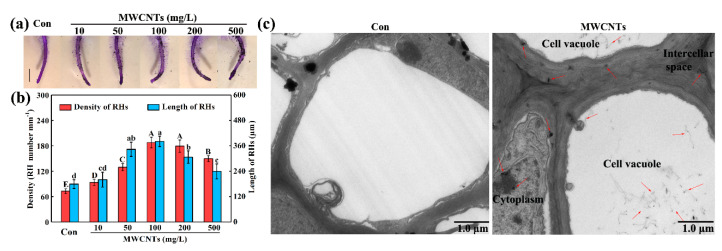
MWCNTs induced rapeseed root hair growth and the distribution of MWCNTs. Two-day-old seedlings were treated with the indicated concentrations of MWCNTs for 3 days. The sample without chemicals was the control (Con). Afterward, photographs of root hairs were taken after staining with 1% toluidine blue (**a**). Scale bar = 1 mm. Meanwhile, the root hair (RH) density (**b**; **left**) and length (**right**) were measured. Within each set of experiments, bars with different letters are significantly different at *p* < 0.05 according to Duncan’s multiple range test. The distribution of MWCNTs was detected in rapeseed root tissues in response to 100 mg/L MWCNTs (**c**). The red arrows point to MWCNTs.

To provide the physiological effects of MWCNTs in root hair development, two-day-old rapeseed seedlings were treated with different concentrations of MWCNTs for 3 days. Compared with the control (Con), 10, 50, 100, 200, and 500 mg/L MWCNTs could differentially promote root hair development (Figure 1a,b). Among these treatments, the response of 100 mg/L MWCNTs was maximal, and this concentration was subsequently used. High concentrations (2000 and 5000 mg/L) of MWCNTs were also applied in the study. The results showed that the root hair development could be seriously impaired by 2000 and 5000 mg/L MWCNTs, while no such significant decrease was found in the presence of 1000 mg/L MWCNTs (Supplementary Figure S1). Seven species, including rice (*Oryza sativa* L.), tomato (*Solanum lycopersicum* L.), Chinese cabbage (*Brassica chinensis* L.), wheat (*Triticum aestivum* L.), radish (*Raphanus sativus* L.), alfalfa (*Medicago sativa* L.), and *Arabidopsis* (*Arabidopsis thaliana* L.), were used subsequently. As shown in Supplementary Table S1, MWCNTs with appropriate concentrations can promote root hair growth, lower and higher concentrations of MWCNTs normally had no such significant effect on root hairs or even inhibited their growth.

The distribution of MWCNTs was further detected by using transmission electron microscopy. The result showed that the MWCNTs were distributed in the cytoplasm, intercellular space, and cell vacuole of rapeseed root tissues, which are highlighted by the red arrows in Figure 1c.

### 2.2. Ethylene Was Involved in MWCNTs-Induced Root Hair Development

**Figure 2:**
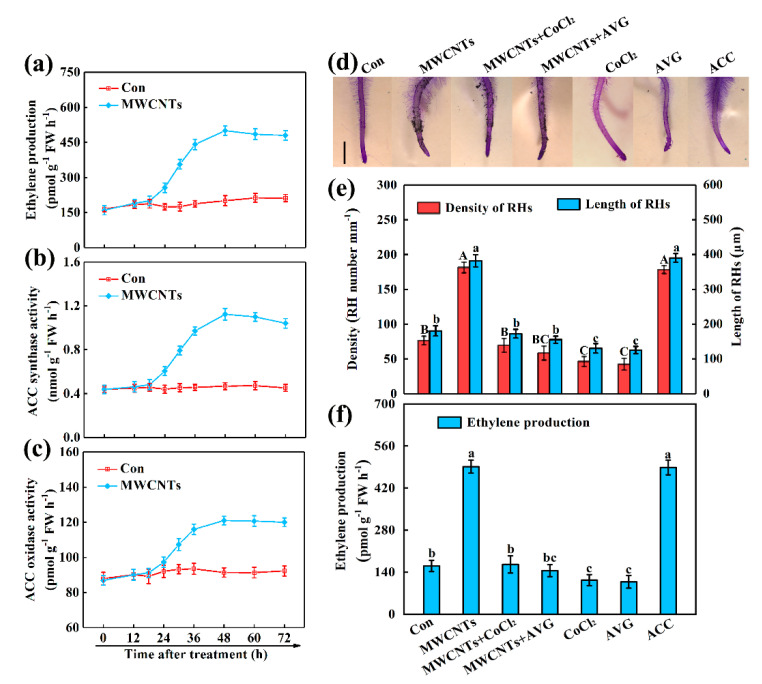
MWCNTs-induced ethylene production and root hair development were blocked by the removal of endogenous ethylene. Two-day-old rapeseed seedlings were treated with 100 mg/L MWCNTs, 10 μM CoCl_2_, and 3 μM AVG, alone or in combinations. The sample without chemicals was the control (Con), and 3 μM ACC alone was used as a positive control. Afterward, the time-course changes in ethylene production (**a**), ACC synthase (**b**), and ACC oxidase activities (**c**) were detected (in control and MWCNTs treated samples). After treatment for 3 days, corresponding photographs of the seedling’s roots were done (**d**), and the root hair (RH) density and length (**e**), and ethylene production (**f**) were determined. Scale bar = 1 mm. Within each set of experiments, bars with different letters were significantly different at *p* < 0.05, according to Duncan’s multiple range test.

In order to assess whether ethylene participated in the MWCNTs-induced rapeseed root hair formation, we measured the contents of endogenous ethylene in root tissues by using gas chromatography. In comparison with the control sample, the time course analysis illustrated that the administration of MWCNTs for 18 h caused a progressive increase in the ethylene production, followed by peaking at 48 h and keeping a stable level until 72 h (Figure 2a). Meanwhile, two critical enzymes for ethylene synthesis, 1-aminocyclopropane-1-carboxylate (ACC) synthase, and ACC oxidase were also analyzed. Similar tendencies were observed in the above enzymatic activities (Figure 2b,c).

Two ethylene synthesis inhibitors, cobalt chloride (CoCl_2_) and aminoethoxyvinylglycine (AVG), were subsequently used. ACC, an ethylene synthesis precursor, was used as a positive control. The results showed that 10 μM CoCl_2_ or 3 μM AVG could not only impaired root hair growth (Figure 2d,e) but also decreased ethylene content (Figure 2f). The MWCNTs-induced root hair development was similar to 3 μM ACC, and it was inhibited by CoCl_2_ or AVG (Figure 2d,e). Meanwhile, the ethylene-induced by MWCNTs was significantly inhibited by CoCl_2_ or AVG (Figure 2f).

### 2.3. NR-Dependent NO Was Associated with MWCNTs-Induced Root Hair Development

**Figure 3:**
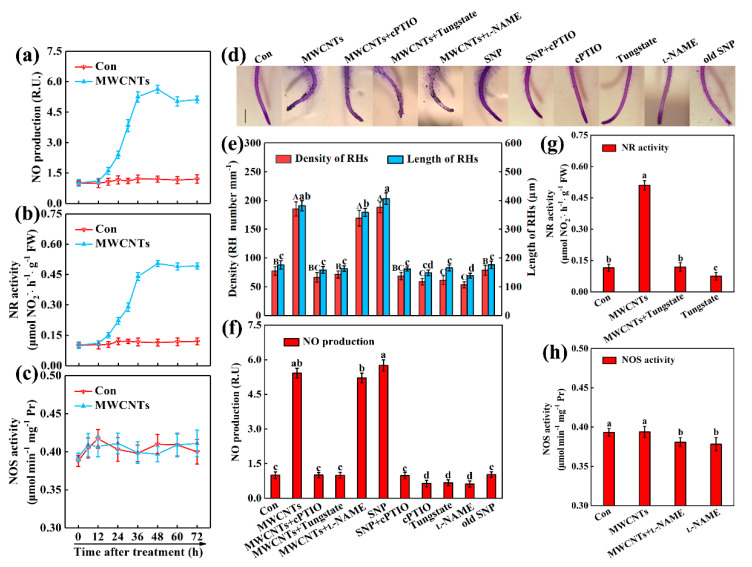
Nitric oxide production and root hair growth induced by MWCNTs were sensitive to cPTIO and tungstate. Two-day-old rapeseed seedlings were treated with 100 mg/L MWCNTs, 20 μM SNP, 100 μM cPTIO, 100 μM tungstate, and 100 μM _L_-NAME, alone or their combinations. The sample without chemicals was the control (Con), and 20 μM old SNP was used as the negative control of SNP. Afterward, the time-course changes in NO production (**a**), NR (**b**), and NOS activities (**c**) were detected (in control and MWCNTs treated samples). After treatments for 3 days, corresponding photographs (**d**), the root hair (RH) density and length (**e**), NO production (**f**), NR (**g**), and NOS activities (**h**) were provided or determined. Scale bar = 1 mm. Within each set of experiments, bars with different letters are significantly different at *p* < 0.05, according to Duncan’s multiple range test.

NO is another gaseous signal molecule in root hair development. In order to evaluate a possible interaction between NO and MWCNTs in root hair development, the endogenous NO signal in rapeseed roots was firstly detected by 4-amino-5-methylamino-2′,7′-difluorofluorescein diacetate (DAF-FM DA). As shown in Figure 3a, compared to the control samples, the fluorescence was significantly induced in the presence of MWCNTs, which showed the initial increase as early as 12 h, and reached a peak at 48 h after treatment (Figure 3a, Supplementary Figure S2). In the time course, experiments found that in the presence of MWCNTs, the activities of NR showed similar tendencies, as compared to the levels of NO production (Figure 3b). However, no significant difference was observed in NOS-like enzyme activities of rapeseed seedlings with or without MWCNTs treatments (Figure 3c).

NO-releasing compound SNP, NR inhibitor tungstate, mammalian NOS inhibitor *N*^G^-nitro-_L_-arginine methyl ester hydrochloride (_L_-NAME), and NO scavenger 2-(4-carboxyphenyl)-4,4,5,5-tetramethylimidazoline-1-oxyl-3-oxiden potassium salt (cPTIO), were used in the subsequent experiments. Old SNP, containing no NO, but nitrate and ferrocyanide was used as a negative control of SNP. The results showed that when tungstate or _L_-NAME was applied alone, both root hair growth (Figure 3d,e) and NO production (Figure 3f) were simultaneously abolished. Meanwhile, old SNP failed to alter root hair development as well as NO production. Both MWCNTs- and SNP-promoted endogenous NO production and root hair development could be respectively blocked by cPTIO. cPTIO alone also resulted in decreased NO content and a significant reduction in root hair growth. In the presence of tungstate, MWCNT-induced NR activities, NO production, and root hair growth were significantly impaired (Figure 3d–g). MWCNTs had no obvious effects on NOS-like activities (Figure 3), and _L_-NAME did not influence MWCNTs-induced NO (Figure 3f) and root hair development (Figure 3d,e).

### 2.4. Ethylene Acts Downstream of NO in MWCNTs-Induced Root Hair Growth

**Figure 4:**
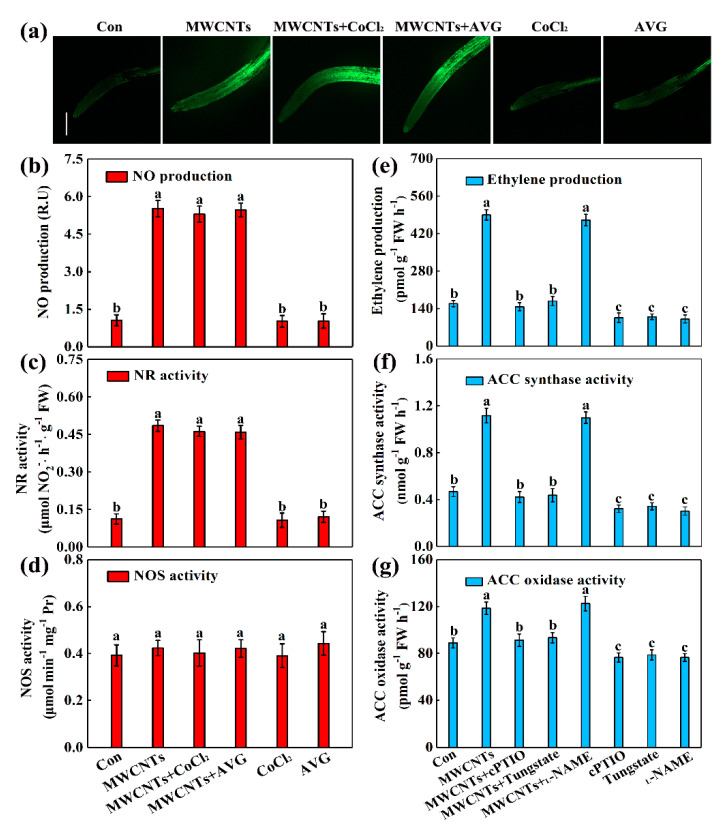
Cross-talk between NO and ethylene. Two-day-old rapeseed seedlings were treated with 100 mg/L MWCNTs, 10 μM CoCl_2_, 3 μM AVG, 100 μM cPTIO, 100 μM tungstate, and 100 μM _L_-NAME, alone or in combinations for 48 h. Afterward, endogenous NO levels (**a**) were determined by laser scanning confocal microscopy, and the corresponding fluorescence densities (**b**), NR (**c**), and NOS activities (**d**) were detected. Scale bar = 0.2 mm. Meanwhile, ethylene production (**e**), ACC synthase (**f**), and ACC oxidase activities (**g**) were also detected. The sample without chemicals was the control (Con). Bars with different letters are significantly different at *p* < 0.05, according to Duncan’s multiple range test.

The interaction between ethylene and NO was also examined in this study. The results showed that two ethylene synthesis inhibitors, CoCl_2_ and AVG, did not alter NO synthesis, including its content, NR, and NOS activities, under the conditions in the presence and absence of MWCNTs (Figure 4a–d). Both cPTIO and tungstate could obviously block ethylene synthesis triggered by MWCNTs, but _L_-NAME had little effect on it. Meanwhile, exogenously applied cPTIO, tungstate, or _L_-NAME alone could decrease ethylene content, and inhibit ACC synthase and oxidase activities (Figure 4e–g).

### 2.5. MWCNTs-Modulated Transcripts Related to Root Hair Development were Dependent on NO and Ethylene Synthesis

**Figure 5:**
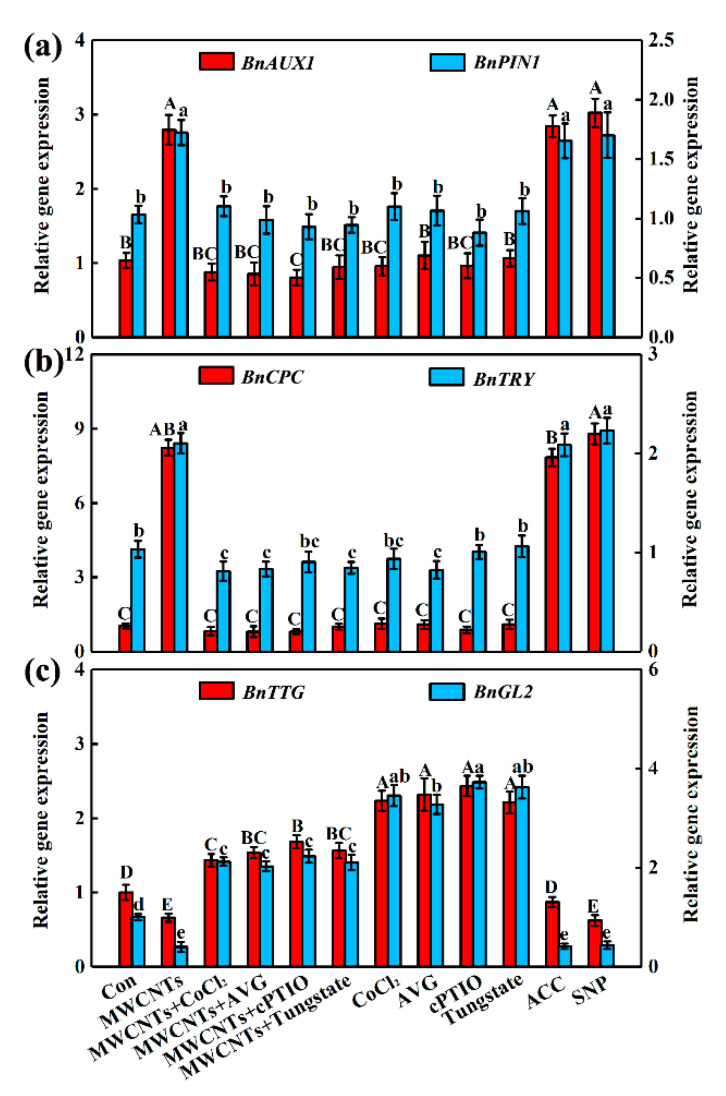
The transcripts levels of root hair development-related genes. Two-day-old rapeseed seedlings were treated with 100 mg/L MWCNTs, 10 μM CoCl_2_, and 3 μM AVG, 100 μM cPTIO, 100 μM tungstate, 3 μM ACC, and 20 μM SNP, alone or in combinations for 24 h. Afterward, *BnAUX1* and *BnPIN1* (**a**), *BnCPC* and *BnTRY* (**b**), and *BnTTG* and *BnGL2* (**c**) transcriptional levels were analyzed by qPCR. The sample without chemicals was the control (Con). Within each set of experiments, bars with different letters are significantly different at *p* < 0.05, according to Duncan’s multiple range test.

The transcripts of auxin signal-related genes *AUXINRESISTANT1* (*AUX1*) and *PIN-FORMED1* (*PIN1*) and root hair development-related genes *TRANSPARENT TESTA GLABRA* (*TTG*), *GLABRA2* (*GL2*), *CAPRICE* (*CPC*), and *TRIPTYCHON* (*TRY*) were quantified by qPCR. Like the responses of ACC and SNP, the expression of *BnAUX1*, *BnPIN1*, *BnCPC*, and *BnTRY* were up-regulated in MWCNTs-induced rapeseed root hair growth, and the expression of *BnTTG* and *BnGL2* were down-regulated (Figure 5). The above responses could be differentially blocked or impaired by the addition of CoCl_2_, AVG, cPTIO, and tungstate (Figure 5).

### 2.6. Genetic Evidence Revealed that Ethylene and NR-Dependent NO Were Associated with MWCNTs-Induced Root Hair Development

To complement the above results, the genetic mutants of *Arabidopsis* were subsequently used. The mutants used in the experiment were NO related mutant *nia1/2* (exhibited impaired nitrate reductase activity) and *noa1* (encoding NO-associated protein 1; with indirectly reduced NO level in vivo), and ethylene related mutant *ein2-5* (ethylene-insensitive mutant) and *ein3-1* (ethylene-insensitive mutant).

**Figure 6:**
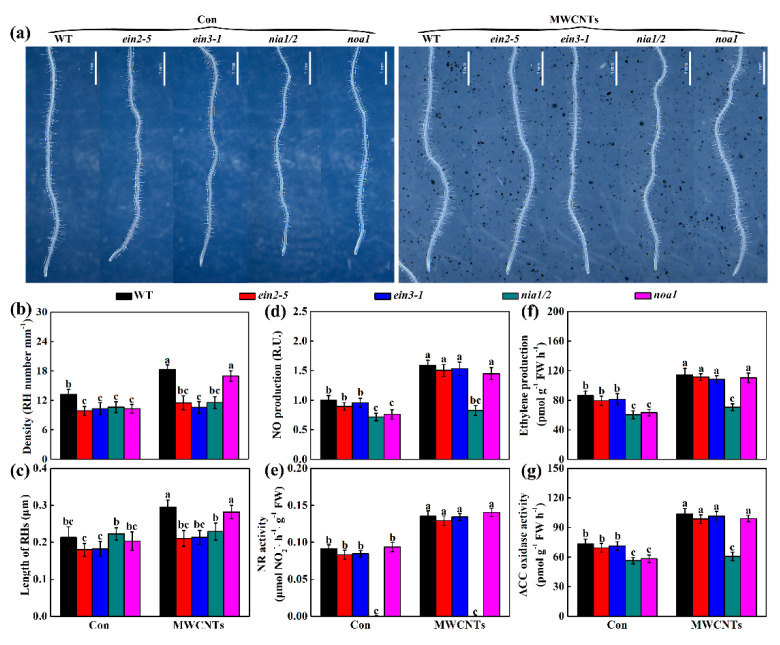
The involvement of NO and ethylene in MWCNTs-triggered root hair development in *Arabidopsis*. Five-day-old WT, *ein2-5*, *ein3-1*, *nia1/2*, and *noa1* mutant plants were grown on MS medium for 5 d and then transplanted to a medium with or without 10 mg/L MWCNTs. After treatments for 5 d, corresponding photographs (**a**), the root hair (RH) density (**b**), and length (**c**) were provided and determined. NO content (**d**), NR activity (**e**), ethylene content (**f**), and ACC oxidase activity (**g**) were also detected after treatments for 3 days. Bars with different letters are significantly different at *p* < 0.05, according to Duncan’s multiple range test.

Lower root hair density in these four mutants and shorter root hairs in *ein2-5* and *ein3-1* were detected in this study (Figure 6a–c). For *nia1/2* and *noa1* mutants, under the control condition, the obvious reduction in NO and ethylene contents were observed (Figure 6d,f), matched with the phenotypes of the root hairs, compared to the wild type (WT) plants (Figure 6a–c). The biochemical analysis further showed that no NR activity was detected in *nia1/2* mutant, normal NR activity was detected in *noa1* mutant (Figure 6e), but a similar and significant reduction in NO content was observed in two mutants (Figure 6d). Meanwhile, no such significant decreases in the NO and ethylene signals were observed in *ein2-5* and *ein3-1* mutants, and the decreased ethylene contents were also observed in *nia1/2* and *noa1* mutants (Figure 6e,g).

Subsequent experiments discovered that the application of MWCNTs could induce root hair growth in WT and *noa1* mutants. Consistently, a significant reduction in root hair development was found in *nia1/2* mutant when challenged with MWCNTs (Figure 6a–c). A similar phenomenon was observed in *ein2-5* and *ein3-1* mutants. Related ethylene and NO synthesis were also examined in the above materials. As anticipated, changes in ethylene and NO contents matched with phenotypes, showing higher ethylene and NO contents in MWCNTs-treated WT, *noa1*, *ein2-5*, and *ein3-1* mutants, which differ from the impaired ethylene and NO contents in *nia1/2* mutant (Figure 6d,f).

The cross-talk between ethylene and NO was further investigated. As shown in Supplementary Figure S3, by using tungstate, an inhibitor of NR, and AVG, an inhibitor of ACC synthetase, we observed that the removal of the major known sources of NO or ethylene severely impaired MWCNTs-induced ethylene production and thereafter root hair formation.

**Figure 7:**
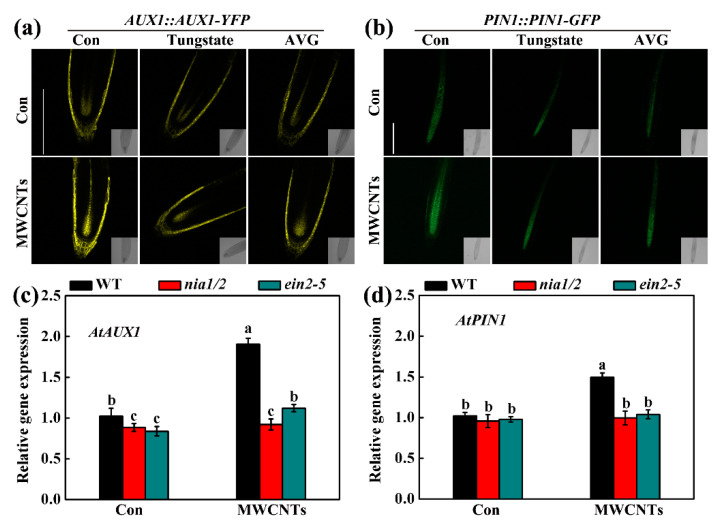
Changes in *AUX1* and *PIN*. Five-day-old *Arabidopsis* seedlings were treated with 10 mg/L MWCNTs, 50 μM tungstate, and 2 μM AVG, alone or their combinations for 24 h. Afterward, the YFP fluorescence images of *AUX1::AUX1-YFP* (**a**) and the GFP fluorescence images *PIN1::PIN1-GFP* (**b**) roots were detected by laser scanning confocal microscopy. Scale bar = 0.2 mm. The mRNA expression of *AtAUX1* (**c**) and *AtPIN1* (**d**) in root tissues were analyzed by qPCR. Bars with different letters are significantly different at *p* < 0.05, according to Duncan’s multiple range test.

**Figure 8:**
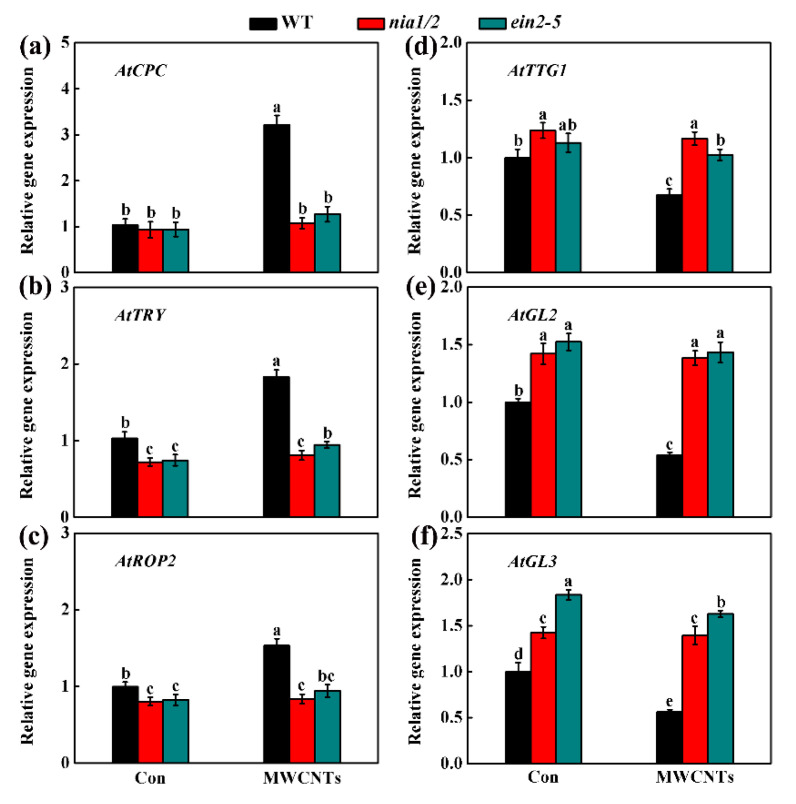
Changes in the transcripts of genes related to root hair formation in *Arabidopsis*. Five-day-old WT, *nia1/2,* and *ein2-5* mutants were grown on MS medium for 5 d, and then transplanted to medium with or without 10 mg/L MWCNTs for 24 h. Seedlings grown on MS medium without MWCNTs were the control (Con). Afterwards, the mRNA expression of *AtCPC* (**a**), *AtTRY* (**b**), *AtROP2* (**c**), *AtTTG1* (**d**), *AtGL2* (**e**), and *AtGL3* (**f**) in root tissues were analyzed by qPCR. Bars with different letters are significantly different at *p* < 0.05, according to Duncan’s multiple range test.

Here, we further evaluated the roles of auxin signaling in MWCNTs response, and found that YFP and GFP fluorescence in the roots of *AUX1::AUX1-YFP* and *PIN1::PIN1-GFP* transgenic plants were increased by MWCNTs, both of which could be differentially impaired in the presence of either tungstate or AVG (Figure 7a,b). The changes of *AtAUX1* and *AtPIN1* transcripts levels were also confirmed by using qPCR in WT, *nia1/2*, and *ein2-5* under normal or MWCNTs-treated condition, showing the decreasing tendencies in two mutants, especially upon MWCNTs (Figure 7c,d). Furthermore, root hair growth-related genes, including *AtCPC*, *AtTRY*, *AtROP2*, *AtTTG1*, *AtGL2*, and *AtGL3*, were also analyzed. Compared to the WT in either the control condition or in the presence of MWCNTs, the down-regulated *AtCPC* (except in the control conditions), *AtTRY* and *AtROP2*, and the up-regulated *AtTTG1*, *AtGL2*, and *AtGL3* in both *nia1/2* and *ein2-5* mutants were observed (Figure 8).

## 3. Discussion

MWCNTs could affect plant growth and stress resistance. However, few studies have focused on root hairs, and their conclusions are controversial. A previous study showed that MWCNTs (diameters ranging from 13 to 14 nm) could significantly promote root hair growth in 70, 80, and 90 mg/L in wheat [21]. However, a controversial result was reported by García-Sánchez et al. [22], which revealed that 25 mg/L COOH-MWCNTs (diameters ranging from 4 to 12 nm; and the modification increases the solubility of MWCNTs) could obviously inhibit root hair growth in *Arabidopsis*. To provide a more detailed analysis of the physiological effects of MWCNTs in root hair development, different concentrations of MWCNTs were used to treat rapeseed (Figure 1a,b and Supplementary Figure S1) and seven other species, including rice, tomato, Chinese cabbage, wheat, radish, alfalfa, and *Arabidopsis* (Supplementary Table S1). The results revealed that MWCNTs could differentially influence the growth of root hairs depending on concentration. MWCNTs with appropriate concentrations can promote plant root hair growth in the species analyzed. Further results showed that the MWCNTs were distributed in the cytoplasm, intercellular space, and cell vacuole of rapeseed root tissues (Figure 1c), consistent with the previous study [13]. These results could provide a basis for the toxicity study of MWCNTs.

In most plants, ethylene signaling is indispensable in root hair development [25,26,27,28,29]. In this study, the time course analysis illustrated that ethylene production could be induced by MWCNTs in rapeseed seedling roots (Figure 2a). Meanwhile, similar tendencies were observed in ACC synthase and ACC oxidase (Figure 2b,c). Ethylene synthesis, in response to MWCNTs in rapeseed, also corresponded with the biological response of MWCNTs control of root hair development (Figure 1a,b). These results inferred that ethylene may be involved in MWCNTs-induced root hair growth. Subsequently, ethylene synthesis inhibitors CoCl_2_ [29,30] and AVG [30,35] were applied to individually decrease ACC oxidase and its synthetase activities, not only impaired root hair growth (Figure 2d,e), but also decreased ethylene content (Figure 2f). The above results clearly confirmed the important function of endogenous ethylene in root hair development, consistent with previous studies [25,26,27,28]. Further results in this study revealed that MWCNTs control of root hair development was similar to the induction role of 3 μM ACC. By contrast, MWCNTs-induced root hair growth was inhibited by CoCl_2_ or AVG (Figure 2d,e). Meanwhile, CoCl_2_ or AVG inhibition of MWCNTs-triggered ethylene was also observed (Figure 2f), indicating a requirement for ethylene in MWCNTs control of root hair development. Similar results were discovered in the previous studies, showing that the removal of endogenous ethylene could impair root hair growth [25,26], since ethylene is an essential signal for controlling root hair development in either nutrient-adequate conditions [26,28] or nutrient-starvation surroundings [27,34].

Similar to ethylene [25,26,27,28], NO is another important signal molecule in root hair development [31,32]. Figure 3a proved that NO was significantly induced by MWCNTs, showing the initial increase as early as 12 h, and reaching a peak at 48 h after treatment (Figure 3a, Supplementary Figure S2). The results clearly suggest that MWCNTs-induced NO is an early event. NR and NOS-like protein are two important enzymes responsible for synthesizing NO in plants [39,40]. From the detection of NR and NOS-like pathways in MWCNTs-treated samples (Figure 3b,c), we inferred that NR was the main enzymatic route for MWCNTs-induced NO. This is consistent with previous studies no matter in plant response against stress [13] or lateral root formation [41]. NR inhibitor tungstate [42,43], mammalian NOS inhibitor _L_-NAME [42,44], and NO scavenger cPTIO [42,45], were used in the subsequent experiments. The results showed that both root hair growth (Figure 3d,e) and NO production (Figure 3f) were simultaneously abolished by tungstate, l-NAME, or cPTIO, emphasizing the important function of endogenous NO in root hair morphogenesis like previous studies [31,32]. In the presence of tungstate, MWCNT-induced NR activities were also significantly impaired (Figure 3g), thus leading to a decreased NO production (Figure 3f) and a reversed phenotype (Figure 3d,e). Comparatively, MWCNTs had no obvious effects on NOS-like activities (Figure 3), and l-NAME did not influence MWCNTs-induced NO (Figure 3f) and root hair development (Figure 3d,e). Those results thus suggested that NOS might not be the main source for NO production elicited by MWCNTs. Similar phenomena were reported in other plant species, including *Arabidopsis* [46], red kidney bean [47], and barley [48]. Combining the above mentioned results, we further proved that MWCNTs-induced root hair development is dependent on NR-mediated NO synthesis.

Ample evidence revealed that NO and ethylene are key signaling molecules participating in various plant signal transduction processes. For example, both ethylene [25,26] and NO [31,32,33,34] are individually suggested to induce root hair growth. However, the question of the relationship between ethylene and NO in MWCNTs-elicited root hair growth appears to be particularly interesting. As shown in Figure 2a and Figure 3a, MWCNTs application stimulated the synthesis of NO and ethylene, and the initial inducible time points were 12 h and 18 h, indicating that NO might act upstream of ethylene in MWCNTs-triggered root hair development. Subsequent experiments further proved that NO is upstream of ethylene by detecting the effects of two ethylene synthesis inhibitors CoCl_2_ and AVG on the NO content induced by MWCNTs; and the effects of the NO scavenger cPTIO, its synthesis inhibitors tungstate and _L_-NAME, on the ethylene content induced by MWCNTs (Figure 4). So, by combining data from Figure 2, Figure 3 and Figure 4, we concluded that some linearity may exist in NO and ethylene signaling downstream of MWCNTs. Interestingly, a similar relationship of NO and thereafter ethylene signaling was observed in the adaptive response of phosphorus-deficient rice [35]. The interaction relationship between NO and ethylene was found in a magnesium-deficiency condition [27]. Therefore, it could be suggested that plant response to an external stimulus is controlled by a complex array of signaling mechanisms, and plants respond differently to various environmental stimuli with the same signals, in a linear fashion or a cross-talk manner.

It is well documented that the transcripts of *TRANSPARENT TESTA GLABRA* (*TTG*), *GLABRA2* (*GL2*), and *GLABRA3* (*GL3*) could promote the development of epidermal cells to nonhair cells, thus resulting in the inhibition in the initiation of root hair development [49,50]. The *Rho-related GTPase from plants* (*ROP*) is closely associated with root hair initiation and tip growth [51,52]. Correspondingly, the transcripts of *CAPRICE* (*CPC*) and *TRIPTYCHON* (*TRY*) are known to trigger root hair formation [49,50]. Auxin signal-related genes *AUXINRESISTANT1* (*AUX1*) and *PIN-FORMED1* (*PIN1*) participate in ethylene- and NO-elicited root hair formation via regulating corresponding marker genes related to root hair development [34,53,54]. Accordingly, the transcripts levels of the above molecular marker genes related to root hair growth were detected (Figure 5). The results indicate that both ethylene and NO are required to modulate these molecular marker genes in MWCNTs control of root hair development. Similarly, previous results also revealed that the genes related to root hair growth were regulated in magnesium deficiency-promoted root hair growth via ethylene and NO signals [34]. The above results indicated that MWCNTs-induced root hair growth is closely associated with the adjustment of the marker gene expression.

Pharmacological experiments may not fully reflect the true roles of endogenous ethylene and NO signals in root hair development and may have side effects [55]. So, the genetic mutants of *Arabidopsis*, which have high homology to rapeseed [38], were also used in the study. Previous reports discovered that *nia1/2*, *noa1*, *ein2-5*, and *ein3-1* showed poor root hair growth [27,34]. Similar results were obtained in our study, showing lower root hair density in these four mutants, and shorter root hairs in *ein2-5* and *ein3-1* (Figure 6a–c), indicating that endogenous ethylene and NO function in plant root hair growth. While, no such significant decreases of NO and ethylene signals were detected in *ein2-5* and *ein3-1* (Figure 6d–g). These could be explained by the fact that both *ein2-5* and *ein3-1* are ethylene-receptor-related mutants that have little effect on endogenous ethylene [56,57,58,59,60]. Further results showed that the application of MWCNTs could induce root hair growth in WT and *noa1*, not in *nia1/2*. (Figure 6a–c), reflecting the important function of NR and ethylene in the above response. The cross talk between ethylene and NO was investigated by using tungstate, an inhibitor of NR, and AVG, an inhibitor of ACC synthetase. As shown in Supplementary Figure S3, the removal of the major known sources of NO or ethylene severely impaired MWCNTs-induced ethylene production and, thereafter, root hair formation. These further indicated a requirement for ethylene in the above MWCNTs responses. Moreover, since AVG did not affect NO production, we further confirm that ethylene acts downstream of NO signaling in MWCNTs governing root hair morphogenesis.

Auxin, a well-known phytohormone, has an important function in controlling root hair growth [31,34]. Previous genetic evidence discovered that auxin might function downstream of ethylene and NO signaling to promote *Arabidopsis* root hair formation under magnesium deficiency conditions [34]. It is well documented that auxin transport is mediated by AUXINRESISTANT1 (AUX1)/LAX influx carriers and the PIN-FORMED (PIN) efflux carrier family [51,52]. For example, it was documented that AUX1 could increase the efficiency of auxin uptake, thus resulting in the efficient transport of auxin and its accumulation within plant tissues. Here, by detecting YFP and GFP fluorescence in MWCNTs-treated *AUX1::AUX1-YFP* and *PIN1::PIN1-GFP* transgenic plants roots in the presence of either tungstate or AVG (Figure 7a,b), and the transcript levels of *AtAUX1* and *AtPIN1* in WT, *nia1/2*, and *ein2-5* under normal or MWCNTs-treated condition (Figure 7c,d), we inferred that auxin might be controlled by ethylene and NO in MWCNTs control of *Arabidopsis* root hair development. Furthermore, the transcript levels of root hair growth-related representative genes, including *AtCPC*, *AtTRY*, *AtROP2*, *AtTTG1*, *AtGL2*, and *AtGL3* [50,51,52], in WT, *nia1/2*, and *ein2-5* under normal or MWCNTs-treated condition (Figure 8) also pointed out that the molecular maker genes associated with root hair growth could be modulated by MWCNTs via NO-ethylene pathway.

**Figure 9:**
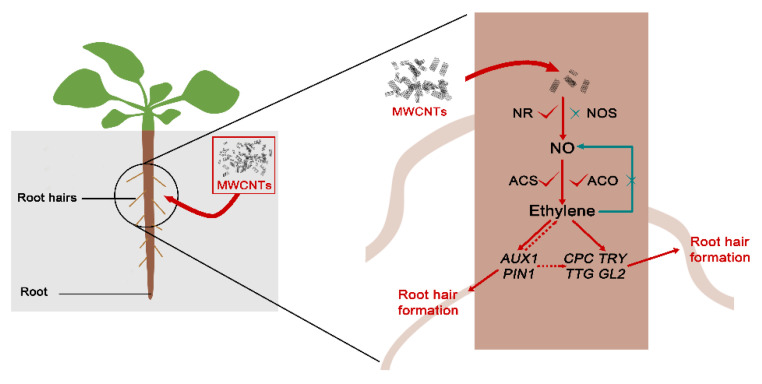
A model depicting the involvement of NO and ethylene in MWCNTs-induced root hair development.

Combining the above results in rapeseed and *Arabidopsis*, we proposed that both ethylene and NO were required for MWCNTs-induced root hair morphogenesis, and ethylene might act downstream of NO in the regulatory cascade. The involvement of auxin signaling was also suggested. A related model is summarized in Figure 9.

## 4. Materials and Methods

### 4.1. Chemicals

All chemicals were obtained from Sigma-Aldrich (St Louis, MO, USA) unless stated otherwise. The chemicals included: multi-walled carbon nanotubes (MWCNTs), 1-aminocyclopropane-1-carboxylic acid (ACC; an ethylene synthesis precursor) [28,30], cobalt chloride (CoCl_2_; an inhibitor of ACC oxidase) [29,30], aminoethoxyvinylglycine (AVG; an inhibitor of ACC synthetase) [29,35], sodium nitroprusside (SNP; a NO-releasing compound) [31,34], 2-(4-carboxyphenyl)-4,4,5,5-tetramethylimidazoline-1-oxyl-3-oxide (c-PTIO; a specific scavenger of NO) [35,36], tungstate (an inhibitor of nitrate reductase) [42,44], *N*^G^-nitro-_L_-arginine methyl ester hydrochloride (_L_-NAME; a mammalian NO synthase inhibitor) [42,43]. Additionally, the old SNP solution, produced by keeping SNP solution for at least 10 d in the light to eliminate the whole NO, was regarded as a negative control of SNP [42,43]. The concentrations of the above chemicals were confirmed in exploratory experiments, from which the maximal effects were confirmed.

The characterization of MWCNTs was carried out in our recent study [13]. They were prepared according to the methods described previously [11,13]. The obtained homogenate colloidal suspension was immediately used.

### 4.2. Plant Materials and Growth Conditions

Rapeseed (*Brassica napus* L. Zhongshuang 11) was purchased in the Chinese Academy of Agricultural Sciences. Seeds were surface-sterilized and cultured according to previous research methods [13]. Briefly, seeds were germinated for 2 days at 25 °C in the darkness, then the identical seedlings were cultured in the 1/2 Murashige and Skoog (MS, pH 5.8) medium at a light intensity of 200 μmol m^−2^ s^−1^ and 16-h/8-h (25 ± 1/23 ± 1 °C) day/night regimes, with the indicated chemicals detailed described in the Figure legends.

*Arabidopsis thaliana* cv. Columbia (Col-0) was used in this study. The wild type (WT), *noa1*, and *nia1/2* mutants were obtained from the *Arabidopsis* Biological Resource Center (http://www.Arabidopsis.org/abrc), and the *ein2-5*, *ein3-1*, *AUX1::AUX1-YFP*, and *PIN1::PIN1-GFP* mutants were generous gifts from C.W. Jin, Zhejiang University, Hangzhou, China.

*Arabidopsis* seeds were surface-sterilized and cultured on the solid 1/2 MS medium containing 1% (*w/v*) agar and 1% (*w/v*) sucrose at 4 °C for 2 days in darkness. Afterward, seeds were grown in a growth chamber at a light intensity of 100 μmol m^−2^ s^−1^ and 16-h/8-h (23/21 °C) day/night regimes. Finally, five-day-old seedlings were then treated with the indicated chemicals, which were detailed described in the Figure legends.

### 4.3. Measurement of Root Hairs

For rapeseed, root hairs were counted and root hair length was measured in the root hair zone of fifteen roots in the third millimeter segment behind the tips by using a microscope (YS100, Nikon, Tokyo, Japan) [61]. Photographs were then taken with a Nikon digital camera (P5000 COOLPIX, Nikon, Tokyo, Japan). For *Arabidopsis*, root hairs were detected as previous described with some modification [27], and fifteen roots were taken for observations carried out on a 5 mm distance from root tip. Additional tests were carried out using rice, tomato, Chinese cabbage, wheat, radish, and alfalfa, and the calculations of their root hair density and length measurements were done as described previously [61].

### 4.4. Detection of MWCNTs Distribution

The distribution of MWCNTs was analyzed by transmission electron microscope as previously described [62]. Six samples from six different rapeseed root tissues (2–3 mm from the tip) per treatment were investigated. For each sample, 6 ultrathin sections were examined by transmission electron microscope (JEM-1400, JEOL, Tokyo, Japan).

### 4.5. Measurement of Ethylene Production, ACC Oxidase, and ACC Synthase Activities

According to methods described previously [27,34], ethylene production, ACC oxidase, and ACC synthase activities in roots was analyzed with a gas chromatograph (GC-7AG; Shimadzu, Tokyo, Japan).

### 4.6. Determination of NO Content, Nitrate Reductase (NR), and NO Synthase (NOS) Activities

According to the previous method [13,27,34], NO in root tissues was visualized using the specific NO probe 4-amino-5-methylamino-2′,7′-difluorofluorescein diacetate (DAF-FM DA) and observed by using a Zeiss LSM 800 confocal microscope (excitation 488 nm, emission 490–530 nm; Carl Zeiss, Oberkochen, Germany). The lower right corners of the photograph were the corresponding brightfield (BF) images. The relative fluorescence was provided as values compared to the control.

According to the previous method [13,41], the NR activity in root tissues was determined spectrophotometrically at 540 nm.

The NOS activity was analyzed using the extinction coefficient of NADPH (6.22 mM^−1^ cm^−1^) [13]. Protein content was also assayed [63].

### 4.7. Analysis of Gene Transcription

RNA isolation and cDNA synthesis were carried out as previously described [13]. Real-time quantitative RT-PCR (qPCR) was conducted, and the gene-specific primers were shown as in Supplementary Tables S2 and S3. Two internal control genes (rapeseed, *BnActin,* and *BnGAPDH*; *Arabidopsis*, *AtActin 2,* and *AtGAPDH*) were used, and the gene expression levels were presented as values compared to the corresponding control samples. The quantification of the relative transcript levels was calculated using the 2^−ΔΔCT^ method [63].

### 4.8. Experimental Design

All experiments were carried out in a random complete block design. Three independent experiments with at least three replicates for each were carried out, and three replicates included 45 seedlings (15 × 3) each time. At least 30 roots per treatment were used to detected NO fluorescence. To analyze the activities of ACC oxidase, ACC synthase, NR, and NOS activities, and the ethylene content, a specified weight of samples per treatment were used.

### 4.9. Statistical Analysis

Values are means ± standard error (SE). Data were analyzed by one-way analysis of variance (ANOVA), taking *p* < 0.05 as significant according to Duncan’s multiple range test.

## 5. Conclusions

Taken together, by using pharmacology, genetics, and molecular approaches, we discovered the part of the molecular mechanism underlying MWCNTs-induced root hair formation in rapeseed and *Arabidopsis*. The results revealed that ethylene and NR-dependent NO are required for MWCNTs-induced root hair morphogenesis via regulating genes related to root hair development, and ethylene may act downstream of NO in this process.

## Supplementary Materials

Supplementary materials can be found at https://www.mdpi.com/1422-0067/21/23/9109/s1.

## Abbreviations

CNTs	carbon nanotubes
MWCNTs	multi-walled carbon nanotube
NR	nitrate reductase
NO	nitric oxide
ACC	1-aminocyclopropane-1-carboxylic acid
AVG	aminoethoxyvinylglycine
NOS	nitric oxide synthase
SNP	sodium nitroprusside
c-PTIO	2-(4-carboxyphenyl)-4,4,5,5-tetramethylimidazoline-1-oxyl-3-oxide
_L_-NAME	*N*^G^-Nitro-_L_-Arginine Methyl Ester Hydrochloride
WT	wild type
