# Supplementary material for: Multi-Walled Carbon Nanotubes Can Promote Brassica napus L. and Arabidopsis thaliana L. Root Hair Development through Nitric Oxide and Ethylene Pathways
Source: Int J Mol Sci. 2020 Nov 30;21(23):9109. doi: 10.3390/ijms21239109 (PMC7729517; doi:10.3390/ijms21239109)
Supplement: Supplementary file 1 [file ijms-21-09109-s001.pdf]

Supplementary Information

Table S1 Effects of MWCNTs on root hair density (number mm^{-1}) in various plant species

	MWCNTs (mg/L)								
	0	10	50	100	200	500	1000	2000	5000
Arabidopsis (5 d)	14.28±1.16b	18.42±1.79a	10.36±1.14c	8.85±2.03c	5.22±1.17d	4.32±1.13d	3.21±1.01e	-	-
Rice (3 d)	21.34±1.36c	21.45±2.30c	27.58±2.07b	30.32±2.96ab	32.19±3.21ab	33.26±2.85a	30.15±2.47ab	25.67±2.18b	22.32±2.53bc
Tomato (3 d)	45.21±3.23c	44.36±2.19c	51.49±2.44b	60.17±3.09a	55.31±3.27ab	50.37±2.39b	45.26±2.13c	40.21±2.36d	38.54±3.17d
Chinese cabbage (3 d)	14.28±1.27c	18.36±2.06b	20.59±1.36a	17.18±1.97b	11.67±1.08d	10.25±1.15de	8.13±1.56e	-	-
Wheat (3 d)	19.17±1.39c	19.53±2.12c	25.05±1.54b	28.27±1.64a	28.56±2.18a	26.21±1.84ab	22.52±1.71bc	20.39±1.58c	18.83±1.69c
Radish (3 d)	30.28±2.36d	32.92±2.73cd	39.58±3.47bc	47.37±3.96ab	52.63±3.57a	42.29±3.17b	35.26±3.28c	28.32±3.45d	22.16±2.83e
Alfalfa (3 d)	23.29±1.59b	24.03±1.62b	26.27±2.35ab	28.35±2.09a	24.55±2.61ab	22.17±2.53bc	18.21±1.75c	-	-

Table S2 The sequences of primers for qPCR used in rapeseed

Primer name	Accession number	Sequence (5’→3’)
BnActin	LOC106418315	F: CTGACCGTATGAGCAAAG<br>R: CCACCGAACCAGAAGGCAGA
BnGAPDH	LOC106382350	F: TGTGCCAATCTACGAGGGTTT<br>R: TTTCCCGCTCTGCTGTTGT
BnAUX1	LOC106376940	F: CAGGAACATCAGTTTCACAGTG<br>R: ATCTGTGATTTCAGCTGCATTC
BnPIN1	LOC106389334	F: TGTACTACACTTCCTCCTCCAT<br>R: TCGTACATATCTTTGCCGGTGA
BnCPC	LOC106454426	F: CAAGGCCAAAGCTTCGTGTT<br>R: CACCTGTCTCCGACGAGTTT
BnTRY	LOC106444610	F: TCGTAGGCGATAGGTGGGAA<br>R: CACAGCCCAAGAAGGAGGTT
BnTTG	LOC106348252	F: CCTCCCACCAAGCTCATGTT<br>R: TGTTGAGGACGGATACTGGC
BnGL2	LOC106356883	F: ACACAGATGGTGATCGCAGG<br>R: TCTGTCTGCCTCTTGTGCAG

Table S3 The sequences of primers for qPCR used in Arabidopsis

Primer name	Accession number	Sequence (5'→3')
AtActin 2	AT3g18780	F: ACAACCGGTATTGTGCTGGA<br>R: GAAGACGGAGGATGGCATGA
AtGAPDH	AT1g13440	F: TTGGTGACAACAGGTCAAGCA<br>R: AAACTTGTCGCTCAATGCAATC
AtAUX1	AT2G38120	F: CAGCCGCCGCACATG<br>R: ACCCTGACTCGATCTCTCAAAGA
AtPIN1	AT1G73590	F: CGTGGAGAGGGAAGAGTTTA<br>R: AACATAGCCATGCCTAGACC
AtCPC	AT2G46410	F: CGAAGAGGTGAGTAGTATCG<br>R: CGACGCCGTGTTTCATAA
AtTRY	AT5G53200	F: GTCGGTGATAGGTGGGAT<br>R: GACGGTGAGGCTTGGTAT
AtROP2	AT1G20090	F: CCGATCTTGCGGCAGAGATGGCGT<br>R: CTTATCACAAGAACGCGCAACGGTT
AtTTG1	AT5G24520	F: TTCCTTCGATTGGAACGATGTA<br>R: GCAAGTCTTAACAAAGGCGTAT
AtGL2	AT1G79840	F: TCTTTAGAGATGAAGCTCGTCG<br>R: CTGTCTTGTCCCTTGGATAAGT
AtGL3	AT5G41315	F: CAAAACGAGGAAGACGATTCAA<br>R: CAACGCCTGAAGAAGAAGATTC

Figure S1

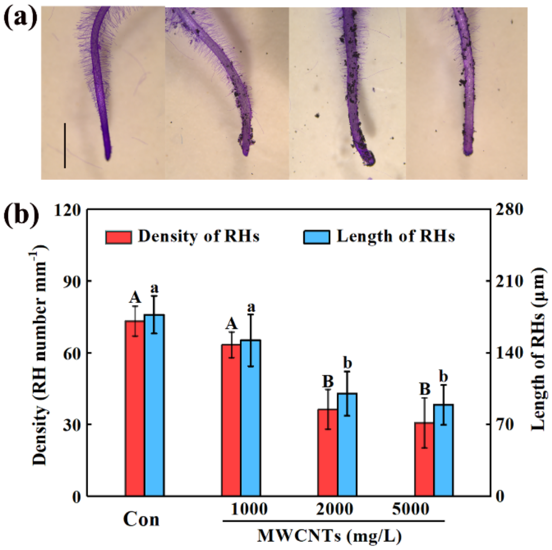

Figure. S1. High concentrations of MWCNTs inhibited rapeseed root hair growth. Two-day-old rapeseed seedlings were treated with indicated concentrations of MWCNTs for 3 days. Afterwards, corresponding photograph of root hairs were taken (a). Scale bar = 1 mm. Meanwhile, the root hair density (b; left) and length (right) were measured. The sample without chemicals was the control (Con). Values are means ± SE of three independent experiments with at least three replicates for each. Within each set of experiments, bars with different letters are significantly different at $P < 0.05$ according to Duncan's multiple range test.

Figure S2

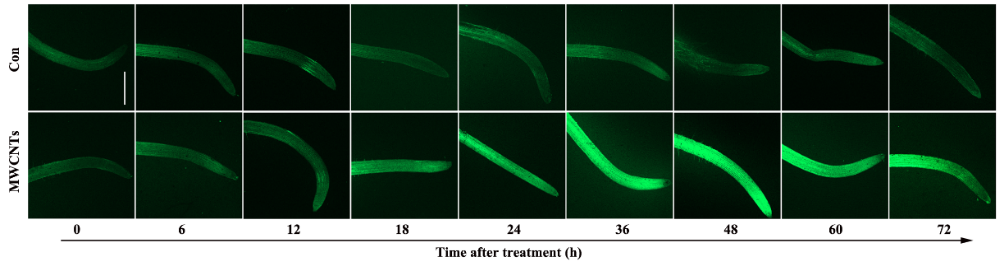

Figure S2. MWCNTs-induced NO. Two-day-old rapeseed seedlings were treated with indicated concentrations of MWCNTs for 3 days. Afterwards, the time-course analysis of DAF-FM-dependent fluorescence in root tissues was provided. Scale bar = 1 mm.

Figure S3

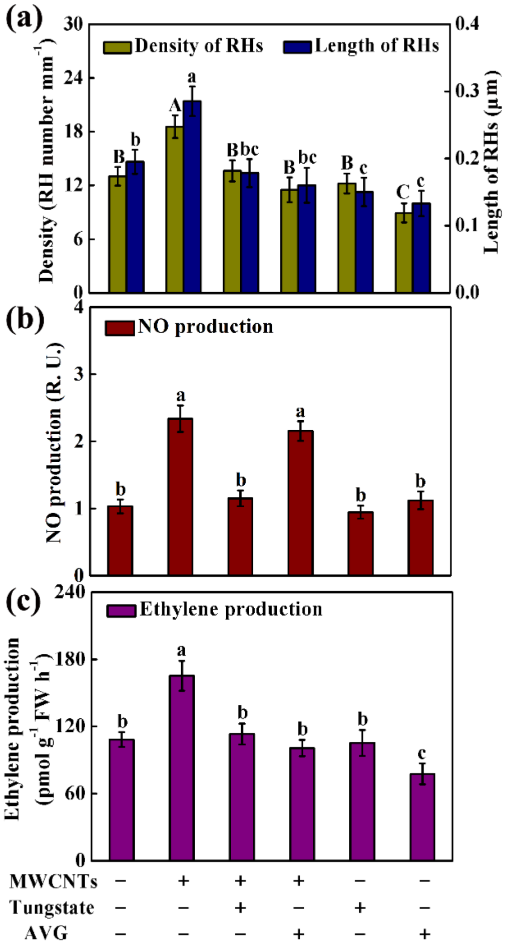

Figure S3. Ethylene might act downstream of MWCNTs induction of NO synthesis in Arabidopsis root hair development. Five-day-old seedlings were treated with 10 mg/L MWCNTs, 50 μM tungstate, and 2 μM AVG, alone or their combinations. After treatments for 5 d, root hair (RH) density (a; left) and length (a; right), NO (b) and ethylene production (c) were detected. Within each set of experiments, bars with different letters are significantly different at $P < 0.05$ according to Duncan's multiple range test.
